# The potential application of concentrated growth factor in pulp regeneration: an in vitro and in vivo study

**DOI:** 10.1186/s13287-019-1247-4

**Published:** 2019-05-20

**Authors:** Fangfang Xu, Lu Qiao, Yumei Zhao, Weiting Chen, Shebing Hong, Jing Pan, Beizhan Jiang

**Affiliations:** 1 Department of Operative Dentistry and Endodontics, School and Hospital of Stomatology, Tongji University, Shanghai Engineering Research Center of Tooth Restoration and Regeneration, 399 Middle Yan Chang Road, Shanghai, 200072 China; 2 Department of Pediatric Dentistry, School and Hospital of Stomatology, Tongji University, Shanghai Engineering Research Center of Tooth Restoration and Regeneration, Shanghai, 200072 China

**Keywords:** Concentrated growth factor (CGF), Human dental stem pulp cells (hDPSCs), Lipopolysaccharide (LPS), Inflammatory microenvironment, Regenerative endodontic treatment (RET)

## Abstract

**Background:**

Concentrated growth factor (CGF), as a natural biomaterial, is known to contain platelets, cytokines, and growth factors to facilitate the healing process, but there has been little information acquired in regenerative endodontics. The purpose of this study was to investigate the effects of CGF on proliferation, migration, and differentiation in human dental stem pulp cells (hDPSCs) exposed to lipopolysaccharide (LPS) in vitro and its potential role in pulp regeneration of the immature teeth in vivo*.*

**Methods:**

In vitro experiments: CGF-conditioned medium were extracted by freeze-dried method. hDPSCs were isolated and identified. The proliferative potential of hDPSCs with different concentration of CGF and LPS was evaluated by Cell Counting Kit-8. Migration capacity was analyzed by Transwell assays, odonto/osteoblastic differentiation was determined by measuring alkaline phosphatase (ALP) activity using ALP staining, and the extent of mineralization was evaluated by using Alizarin red S staining. The mRNA expression level of DMP-1, DSPP, OPN, Runx2, and OCN were determined by quantitative polymerase chain reaction (qPCR).

In vivo experiments: CGF were used as root canal filling agent of the immature single-rooted teeth in the beagle dogs. The teeth were then radiographed, extracted, fixed, demineralized, and subjected to histologic analyses at 8 weeks. The newly formed dentine-pulp complex and the development of apical foramen were evaluated by the hematoxylin-eosin (HE) and Masson trichrome technique. Soft tissues were analyzed by immunohistochemical staining of vascular endothelial growth factor (VEGF) and Nestin.

**Results:**

In vitro experiments: The cultured cells exhibited the characteristics of mesenchymal stem cell. The treatment of LPS significantly increased the expression of TNF-α, IL-1β, IL-6, and IL-8 in hDPSCs, and CGF inhibited the mRNA expression of IL-8 in LPS-stimulated hDPSCs. The proliferation values of the CGF group in LPS-stimulated hDPSCs were significantly higher than that of the control group from day 3 to day 7 (*P* < 0.05). In addition, the number of migratory cells of the CGF group was greater than that of the control group at 24 h with or without LPS treatment. ALP activities increased gradually in both groups from day 4 to day 7. The mineralized nodules and the expression of odontogenesis-related genes DMP-1 and DSPP, osteogenesis-related genes OPN, Runx2, and OCN were dramatically enhanced by CGF in LPS-stimulated hDPSCs at days 21 and 28.

In vivo experiments: In CGF treated group, the results of radiograph, HE, and Masson trichrome staining showed a continuing developed tooth of the immature teeth in the beagle dogs (i.e., the ingrowth of soft tissues into the root canal, the thickened internal root dentin walls, and the closed apex), which resembled the normal tooth development in the positive control group. The immunohistochemical staining showed that VEGF and Nestin were both moderately expressed in the regenerated pulp-like tissues which indicating the vascularization and innervation.

**Conclusions:**

CGF has a positive effect on the proliferation, migration, and differentiation of hDPSCs exposed to LPS in vitro, and it can also promote the regeneration of dentine-pulp complex of the immature teeth in the beagle dogs in vivo. Therefore, CGF could be a promising alternative biomaterial in regenerative endodontics.

## Background

Pulpitis and periapical periodontitis in immature tooth are common diseases in dental clinical treatments that derived from bacterial infection. The emergence of pulp inflammation is caused by the response of dental pulp tissue to foreign bacterial component and its byproducts. Dental pulp tissue has the ability to repair and regenerate, minor inflammation can stimulate dental pulp stem cells reside in the pulp to migrate to the injured site, where they subsequently differentiate into odontoblasts and participate in repairing the dentine-pulp complex [1]. Although current treatment for pulpitis and periapical periodontitis in immature tooth are apexification and apical barrier technique, there still exist many undeniable drawbacks including postoperative fractures and reinfections result from the arrested root development [2]. Therefore, how to facilitate the repairing process of dental tissues under inflammatory microenvironment to achieve pulp regeneration has drawn more attention in current endodontic researches.

As an alternative approach, regenerative endodontic treatment (RET) aims to replace inflamed/necrotic pulp tissue with regenerated pulp-like tissue hereby to achieve the maximum conservation of tooth vitality and the continued development of immature tooth [3]. Three key elements which are essential for tissue regeneration are stem cells, bioactive molecules, and scaffolds. Concentrated growth factor (CGF), which is known as bioscaffolds and a reservoir of cytokines, has been used for bone regeneration in current clinical implantological application [4, 5]. As the latest generation of platelet concentrate products, the modified production process of CGF is simpler and requires repeatedly switching the centrifugation speed [6]. As a result, the relatively stiffer structure of CGF is more similar to a natural fibrin and contains abundant growth factors and proteins from autologous platelets and leucocytes [7]. CGF contains large amounts of growth factors including platelet-derived growth factor-BB (PDGF-BB), transforming growth factor β-1 (TGF-β1), insulin-like growth factor-1 (IGF-1), vascular endothelial growth factor (VEGF), and basic fibroblast growth factor (bFGF). All these factors were intimately involved in the regulation of cell differentiation, proliferation, and angiogenesis which were vital for tissue regeneration [8]. However, it also contains several proinflammatory cytokines including IL-6 and IL-1β that may exert negative effects on tissue repair and regeneration [9].

Gram-negative bacteria species are the most common microorganisms that account for necrotic pulp. Lipopolysaccharides (LPS) are a major molecular component of the cell wall of these bacteria and the prime toxic factor that contribute to the bacterial-induced immune response [10, 11]. In several experiments, LPS has been used to form a model of inflammation [12–14]. Current studies about CGF in tissue regeneration have been mainly focused on bone regeneration [15, 16], and our previous studies have investigated the effects of CGF on human stem cells of the apical papilla (SCAPs) and proved its potentials in regenerative endodontics [17]. Therefore, the purpose of this study was to evaluate the effects of CGF on the proliferation, migration, and differentiation of hDPSCs under LPS-inflamed condition in vitro and its potential role in pulp regeneration of the immature teeth in vivo*.*

## Materials and methods

The present work was developed according to the principles recommended for experimentation with human beings and the animals determined by the Institutional Review Board of Tongji University, and ethics committee approval was obtained (No. 2018-012). All subjects enrolled were informed about the procedures and objectives of the study and signed a consent form.

### Isolation and characterization of hDPSCs

Normal impacted third mandibular molars were collected (*N* = 6, aged 14–20 years) from healthy patients with informed consent in a dental clinic at the Affiliated Stomatology Hospital of Tongji University. In brief, the DPSCs were isolated by enzyme digestion according to a previously described method [18]. The separated cells were cultured in Dulbecco’s modified Eagle’s medium (Hyclone, Logan, UT, USA), supplemented with 10% fetal bovine serum (FBS; Gibco BRL, USA) and 100 U/ml penicillin-G (Sigma, St Louis, MO, USA) and streptomycin in a humidified atmosphere of 5% CO_2_ at 37 °C. Cells at passage 3 (P3) were used to the following study.

The characterization of DPSCs was analyzed by flow cytometry and three multilineage differentiation assays as previously described [19, 20]. In brief, CD105, CD90, and CD146 were used as surface markers of mesenchymal stem cell and CD34 and CD45 were used to confirm that the hDPSCs were not hematopoietic lineage cells (R&D Systems, Minneapolis, MN, USA). The isotype served as the negative control. Each experiment was performed with a BD FACsCalibur (BD Biosciences, San Jose, CA, USA). For the three multi-lineage differentiation assay, alizarin red S staining, alcian blue staining, and oil red staining were used to identify the mineralized nodule, glycosaminoglycans, and lipid droplet after the cells were incubated with a different inducing medium for 3 weeks.

### Conditioned medium preparation

Venous blood (10 mL) was collected from each participant after providing informed consent; the tubes were immediately centrifuged in a special centrifuge device by 30 s acceleration, 2 min at 2700 rpm (600 g), 4 min at 2400 rpm (400 g), 4 min at 2700 rpm (600 g), 3 min at 3000 rpm, and 36 s deceleration (MEDIFUGE™, Silfradentsrl, S. Sofia, Italy). Conditioned medium (CM) was prepared as described previously with slight modifications [17]. In brief, the isolated CGF membranes were frozen overnight in a vacuum freeze dryer. To harvest the cytokines, the lyophilized membrane was pulverized and immersed in 50 mL DMEM. The medium was collected after incubation at 4 °C for 24 h and was centrifuged to remove the red blood cells. CM was completed after being supplemented with 10% fetal bovine serum and 1% antibiotic. Four concentrations of 1 **×** CGF (CGF isolated from 10 mL venous blood dissolved in 50 mL DMEM), 1/2**×**, 1/4**×**, and 1/8**×** CGF were used.

### LPS treatment and the detection of inflammation-related genes

hDPSCs were treated with 0, 0.1, 1, and 10 μg/mL LPS (*Escherichia coli* 0111:B4, Sigma) and/or 1**×** CGF for 24 h. The messenger RNA expression levels of IL-6, IL-8, IL-1β, and TNF-α were determined by quantitative polymerase chain reaction. The cells of the different group were lysed by Trizol reagent (Life Technologies, Carlsbad, CA, USA), and total RNA was isolated. The extracted RNA was reverse transcribed using a complementary DNA synthesis kit (Roche, Schlieren, Switzerland), and the relative messenger RNA expression of the target gene was analyzed using the FastStart Essential DNA Green Master (Roche, Schlieren, Switzerland). Glyceraldehyde-3-phosphate dehydrogenase (GAPDH) was used as the control to normalize the RNA expression levels. The primer sequences for IL-6, IL-8, IL-1β, and TNF-α (Sango Biotech, Shanghai, China) are listed in Table 1.

**Table 1:** Primers sequences used in the real-time PCR

Gene	Forward primer	Reverse primer
IL-6	GGTGTTGCCTGCTGCCTTCC	GTTCTGAAGAGGTGAGTGGCTGTC
IL-8	CAAGCTGGCCGTGGCTCTC	GGTCCACTCTCAATCACTCTCAGTTC
IL-1β	TGGCTTATTACAGTGGCAATGAGGATG	TGTAGTGGTGGTCGGAGATTCGTAG
TNF-α	CGTGGAGCTGGCCGAGGAG	AGGAAGGAGAAGAGGCTGAGGAAC
DSPP	GGAGCCACAAACAGAAGCAACA	TGGACAACAGCGACATCCTCAT
DMP1	CAGGAAGAGGTGGTGAGTGAGT	TGGATTCGCTGTCTGCTTGCT
Runx2	TCCAGACCAGCAGCACTCCATA	TCCATCAGCGTCAACACCATCA
OPN	TGCTACAGACGAGGACATCACC	TCTGGACTGCTTGTGGCTGTG
OCN	CCGCAGCTCCCAACCACAAT	GCCAGCCTCCAGCACTGTTTA
GAPDH	CCAGAACATCATCCCTGCCTCT	GACGCCTGCTTCACCACCTT

### Cell proliferation assay

The hDPSCs were seeded on a 96-well plate (Corning, NY, USA) at a density of 2 **×** 10^3^ cells per well and were cultured with different concentrations of CGF and 0.1 μg/mL, 1 μg/mL, and 10 μg/mL LPS (*E. coli* 0111:B4, Sigma) for 1, 3, 5, and 7 days. The normal medium was used as the control group. The culture medium was replaced with fresh culture medium every 2 days. Cell Counting Kit-8 (Keygen, Nanjing, China) was used to analyze the cell numbers. The optical density values were measured using a microplate reader (BioTek, Winooski, VT) at 450 nm. The results from different groups were compared. After analyzing the results comprehensively and for the sake of research convenience, we selected the concentration of 1**×** CGF for this study.

### Cell migration assay

Twenty-four plates of Transwell filter inserts (Corning, NY, USA) were used to investigate the migratory capacity of hDPSCs after being treated with CGF with or without 1 μg/mL LPS presence at 24 h. Normal media with or without 5% serum served as the positive and negative control groups, respectively. The migrated cells were stained by crystal violet and were counted randomly in six microscope fields. The cell numbers per field were calculated using ImageJ software (version 10.2; National Institutes of Health, Bethesda, MD, USA), and the average was analyzed using GraphPad Prism v4.0 software (GraphPad Software, La Jolla, CA, USA).

### Detection of ALP activity

hDPSCs were cultured with 1 μg/mL LPS, 1**×** CGF, or a combination of LPS and CGF for 4 and 7 days. The culture medium was replaced with fresh culture medium every 2 days. For ALP staining, the media were removed, and the cells were fixed in 70% ethanol for 1 h. After the cells were rinsed three times with deionized water, a 5-bromo-4-chloro-3-indolyl phosphate/nitroblue tetrazolium solution (Beyotime, Shanghai, China) was added to each well. Then, the stained cells were photographed after several steps of washing. For quantitative analysis, the stain was extracted with 10% (*w*/*v*) cetylpyridinium chloride (Sigma-Aldrich) for 15 min, and stain intensity was quantified by measuring the absorbance at 562 nm on an absorbance microplate reader (BioTek, Winooski, VT, USA).

### Alizarin red S staining

Alizarin red S staining was performed to detect the mineralization nodules of the hDPSCs after culture in CGF and/or LPS for 21 and 28 days. In brief, the paraformaldehyde-fixed cells were stained with 0.5% alizarin red S solution (Sigma-Aldrich, St Louis, MO, USA) and were photographed after several steps of washing.

### Real-time quantitative polymerase chain reaction

The messenger RNA expression levels of dentin matrix protein 1 (DMP-1), dentin sialophosphoprotein (DSPP), osteopontin (OPN), Runt-related transcription factor 2 (Runx2), and osteocalcin (OCN) were determined by quantitative polymerase chain reaction. The method was the same as the qPCR procedure mentioned above. The primer sequences for DSPP, DMP-1, OPN, Runx2, and OCN (Sango Biotech, Shanghai, China) are listed in Table 1.

### Orthotopic transplantation assay of immature tooth with CGF in the beagle dogs

The beagle dogs (*N* = 3) approximately 5 months old were obtained from the Experimental Animal Center of Jiagan Biotechnological Limited Company (Shanghai, China). Thirty-six single-rooted anterior teeth were randomly divided into three groups including CGF group (*N* = 12), positive control group (normal teeth with no treatment, *N* = 12), and negative control group (root canal were prepared only, *N* = 12). Preoperative radiographs were obtained to confirm the presence of an open apex and the absence of preexisting pathosis in each canine tooth.

All experimental procedures were conducted under a clean protocol with the use of sterile materials and equipment according to a previously described method [21]. Under general anesthesia induced by Pentothal (Sinopharm Chemical, China) 13.5 mg/kg intravenously and intubation and maintenance with isoflurane (Sinopharm Chemical, China) supplemented with local anesthesia (Alticaine epinephrine; Sinopharm Chemical, China), venous blood (10 mL) was collected from the beagle dog and immediately centrifuged in a special centrifuge device (MEDIFUGE TM, Silfradentsrl, S. Sofia, Italy) as elaborated above and the CGF was then cut into 2 mm^3^ fragment on ice for the later use. The pulp of all experimental teeth was mechanically exposed with a 0.6-mm-diameter diamond burs in a high-speed handpiece. Sterile stainless steel endodontic broaches (Dentsply Tulsa Dental, USA) were used to extract the pulp tissues in the root canals. To avoid teeth impairment from heat during preparation, the teeth and cutting instruments were irrigated with sterile normal saline solution. Root canals were firstly prepared by using the #15 sterile stainless steel K file (Dentsply Tulsa Dental, USA) pull up and down along with the surrounding root canal walls, then by ultrasonic irrigated with 1.25% NaClO and 17% EDTA. Finally, the root canals were irrigated with sterile distilled water. After dried with sterile paper tip, the root canals were directly filled with CGF fragment for CGF group and then rebased with 2 mm BP plus (Innovative Bioceramix, CA, USA). In the negative control group, the pulp cavity was rebased with 2 mm BP plus without any filling material inside the root canal. All cavities were subsequently restored with glass ionomer cement (Fuji IX; GC International Corp, Tokyo, Japan) according to the manufacturer’s instructions. Eight weeks later, the related teeth were radiographed to determine the conditions of root development.

### Sample collection

The animals were killed under general anesthesia provided by Jiagan Biotech Company (pentobarbital; Sinopharm Chemical, China) at 30 mg/kg intravenously. The carotid arteries were exposed and cannulated. The animals were euthanized with additional pentobarbital (Sinopharm Chemical, China) at a dose of 90 mg/kg intravenously. The animals were perfused with 4% paraformaldehyde (Sangon Biotech, Shanghai, China). The involved teeth were extracted and fixed in 4% paraformaldehyde (Sangon Biotech, Shanghai, China) for 24 h at 4 °C. The samples were demineralized in 10% EDTA for 2 months at 37 °C then embedded in paraffin. The sections with a thickness of 5 μm were cut in a mesiodistal direction for HE staining and immunohistochemistry (IHC).

### Histologic evaluation

For the HE and Masson trichrome staining, the slides were deparaffinized and rehydrated by gradient elution using xylene and ethanol, and then were stained by hematoxylin-eosin (Keygen, Nanjing, China) and Masson trichrome staining reagent (Keygen, Nanjing, China) according to the manufacturer’s instructions.

### IHC

For the IHC, the primary antibodies used for immunochemistry were as follows: VEGF (Bioss, bs-1665R) and Nestin (Abcam, ab7659). Secondary antibodies were all purchased from Boster in China. In brief, the slides were deparaffinized and rehydrated by gradient elution using xylene and ethanol, followed by incubation in 3% H_2_O_2_ to suppress endogenous peroxidase activity. For antigen retrieval, the slides were incubated in hyaluronidase (Sigma-Aldrich, St Louis, MO, USA) for 1 h at 37 °C. After the sealing of 5% BSA, the specimens were incubated with primary antibody diluted 1:100 at 4 °C overnight. Then sections were then rinsed in PBS and incubated with biotinylated secondary antibody for 20 min at room temperature. SABC kit purchased from Boster (Wuhan, China) was used for the staining process. 3,3′-Diaminobenzidine (DAB) was used as a color developing agent, and then slides were counterstained with hematoxylin. Finally, slides were mounted with Permount TM Mounting Medium and observed by the stereomicroscope (Carl Zeiss, Stemi 508, Germany) and the microscope (Nikon Eclipse 80i, Japan). Pictures were modified by PhotoshopCS6 (Adobe, CA, USA). Negative controls were incubated with normal anti-rabbit or anti-mouse IgG instead of the primary antibodies.

### Statistical analysis

All experiments were performed in triplicate, and statistical analysis was performed by using SPSS (IBM SPSS, Armonk, NY, USA) version 20.0. Mean values were calculated and presented with an error bar representing ±SD. The one-way analysis of variance (ANOVA) test was used for statistical analysis. Statistical significance was accepted at *P* < 0.05 and graphics software used was GraphPad Prism 6.0.

## Results

### Mesenchymal stem cell characteristics of hDPSCs

**Fig. 1:**
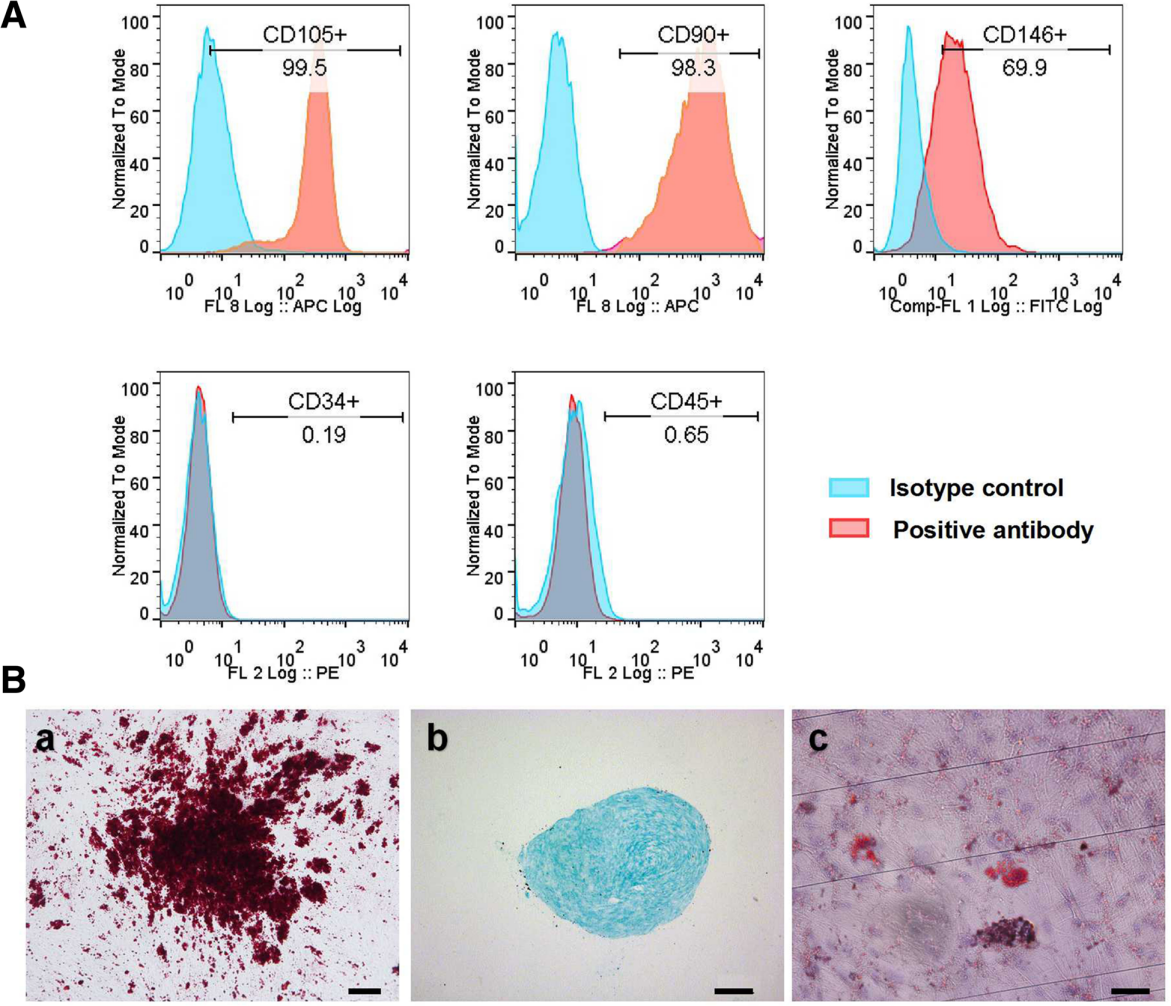
Mesenchymal stem cell characteristics of hDPSCs. **A** hDPSCs expressed surface mesenchymal stem cell markers. The hDPSCs highly expressed the mesenchymal stem cell-related antigen CD105, CD90, and CD146, but they did not express the hematopoietic cell antigen CD34 and CD45. **B** hDPSCs demonstrated the multilineage differentiation capacity. **a** Mineralization nodule formation, positive to alizarin red S staining. **b** Positive staining with alcian blue of glycosaminoglycan is shown. **c** Lipid droplet accumulation by oil red O staining

Cells successfully grew out from the extracted dental pulp tissue after cultured for 3–7 days. The primary cells showed plastic adherence and exhibited a spindle shape. The flow cytometry analysis indicated that hDPSCs expressed the mesenchymal stem cell-related antigen CD90, CD105, and CD146 but did not express the hematopoietic cell antigen CD45 and CD34 (Fig. 1A). hDPSCs also could be induced to three lineages of differentiation; after 3 weeks of osteogenic, adipogenic, and chondrogenic induction, extensive amounts of mineralized nodules, lipid droplets, and glycosaminoglycans were observed with alizarin red, oil red O, and alcian blue staining, respectively (Fig. 1B).

### Consequence of LPS on hDPSCs

**Fig. 2:**
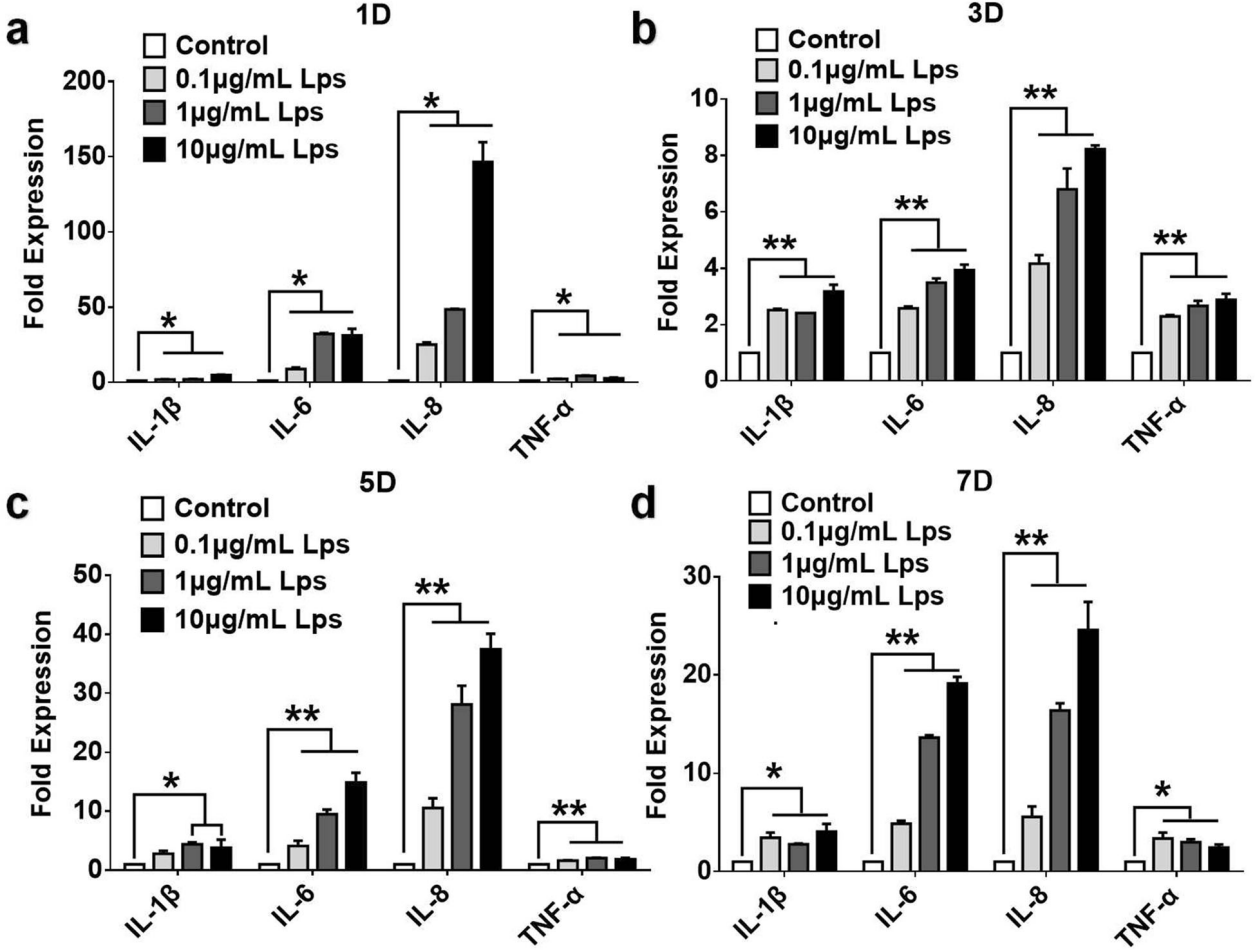
The effects of LPS on the expression of proinflammatory cytokines in hDPSCs. The release of TNF-α, IL-1β, IL-6, and IL-8 of hDPSCs after 1 (**a**), 3 (**b**), 5 (**c**), and 7 (**d**) days incubation of different dose of LPS were determined by qPCR. The results are mean ± standard deviation of triplicate measurements from three independent experiments

After 1, 3, 5, and 7 days of LPS (0.1, 1, 10 μg/mL) treatment, the expression of IL-6, IL-8, TNF-α, and IL-β were all significantly increased and LPS have increased the expression of IL-6 and IL-8 in a dose-dependent manner at days 3, 5, and 7 (Fig. 2).

### Effect of LPS and CGF on hDPSC proliferation

**Fig. 3:**
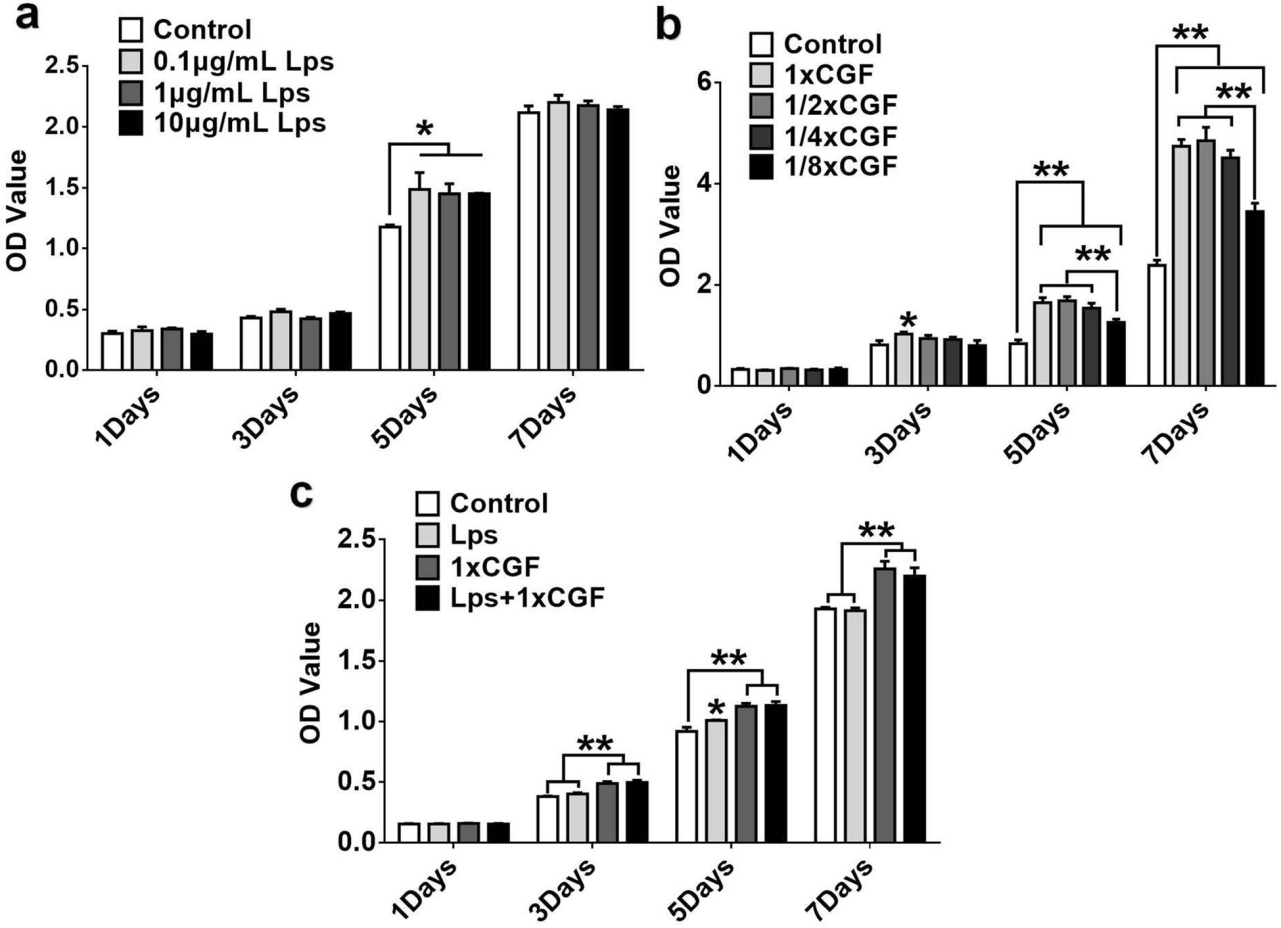
The effects of LPS and CGF on the proliferation of hDPSCs. The proliferation under treatment of different concentrations of LPS (**a**) and CGF (**b**) and both (**c**) was assessed by CCK-8 assay

After 5 days treatment with LPS (0.1, 1, 10 μg/mL), the proliferation ratio of hDPSCs was significantly higher than that of the control group (*P* < .05) (Fig. 3a). 1×, 1/2×, 1/4×, and 1/8× CGF showed no accelerated effect on hDPSC proliferation on day 1. However, 1× CGF increased proliferation ratio of hDPSCs on day 3, and on days 5 and 7, different concentrations of CGF could significantly increase cell proliferation in a dose-dependent manner, and the promoting effect on cell proliferation of 1/8× CGF was less obvious than that in 1×, 1/2×, and 1/4× CGF group (Fig. 3b). When hDPSCs were treated with 1× CGF and 1 μg/mL LPS at the same time, the proliferation ratio was also enhanced as compared with the control group from day 3 to day 7 (Fig. 3c).

### Effects of CGF on the expression of inflammation-related genes in LPS-stimulated hDPSCs

**Fig. 4:**
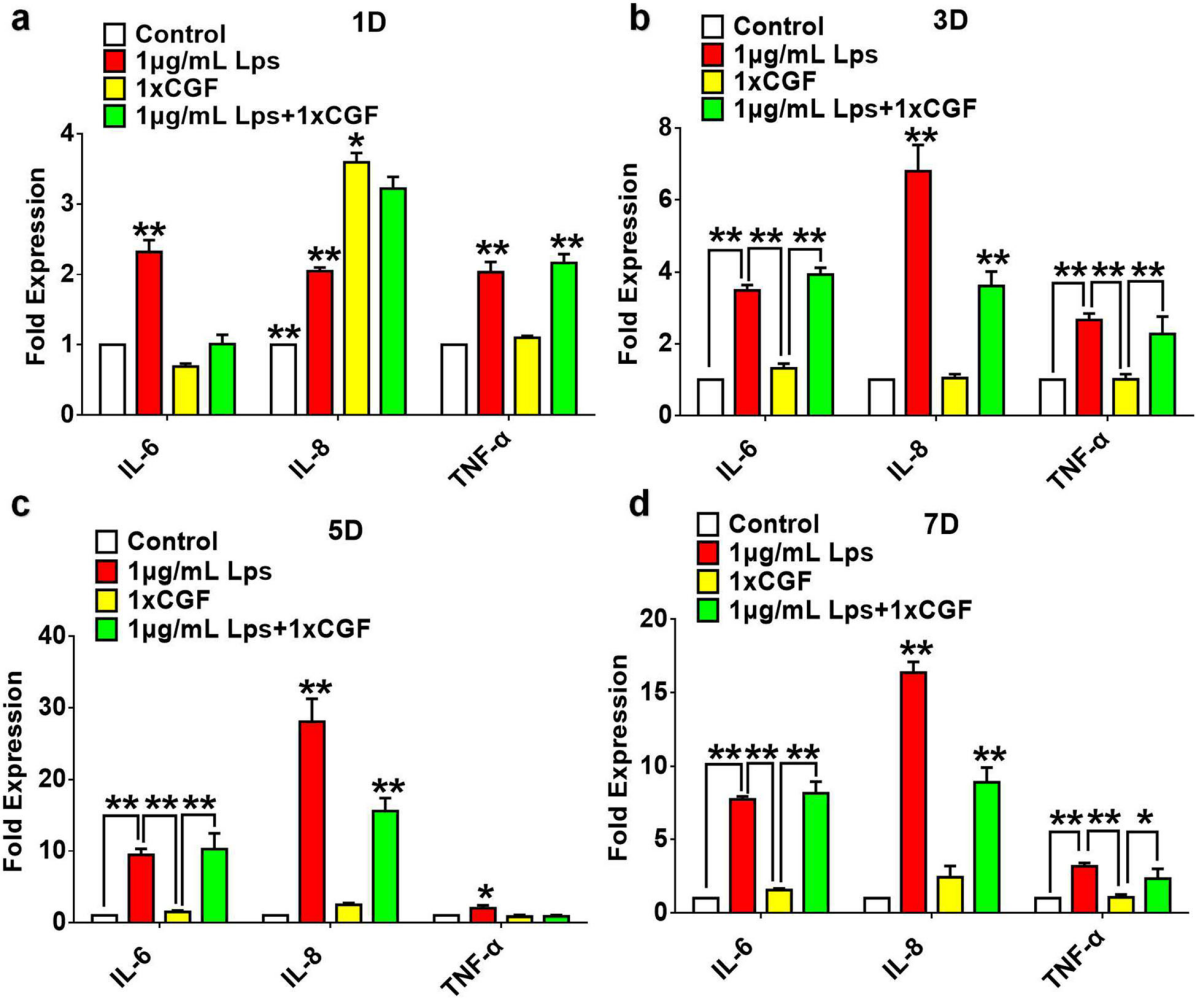
Effects of CGF on the expression of proinflammatory cytokines in LPS-stimulated hDPSCs. Cells were cultured with or without 1**×** CGF in the presence of 1 μg/mL LPS for 1 (**a**), 3 (**b**), 5 (**c**), and 7 (**d**) days. The release of IL-6, IL-8, and TNF-α were determined by means of by qPCR (*P < .05, **P < .01)

To determine the effect of CGF on proinflammatory cytokines, hDPSCs were cultured with or without 1× CGF in the presence of 1 μg/mL LPS for 1, 3, 5, and 7 days and examined by qPCR to detect the release of cytokines including IL-6, IL-8, and TNF-α. The results showed that IL-8 was highly detected in CGF at initial 24 h, and the treatment with CGF can significantly attenuate the LPS-stimulated release of IL-8 in hDPSCs at 1, 3, 5, and 7 days (Fig. 4). Besides, the expression of IL-6 was significantly suppressed by CGF in LPS-stimulated hDPSCs at initial 24 h and then exhibited no significant decrease on that as compared with the LPS treatment group (Fig. 4). Moreover, the expression of TNF-α was upregulated at day 1 after CGF treatment in LPS-stimulated hDPSCs (Fig. 4a), and then slightly inhibited by CGF in LPS-stimulated hDPSCs at days 3 and 7 (Fig. 4b). At day 5, qPCR results demonstrated that CGF significantly decreased the LPS-stimulated release of TNF-α in hDPSCs (Fig. 4c).

### Effect of CGF on cell migration in LPS-stimulated hDPSCs

**Fig. 5:**
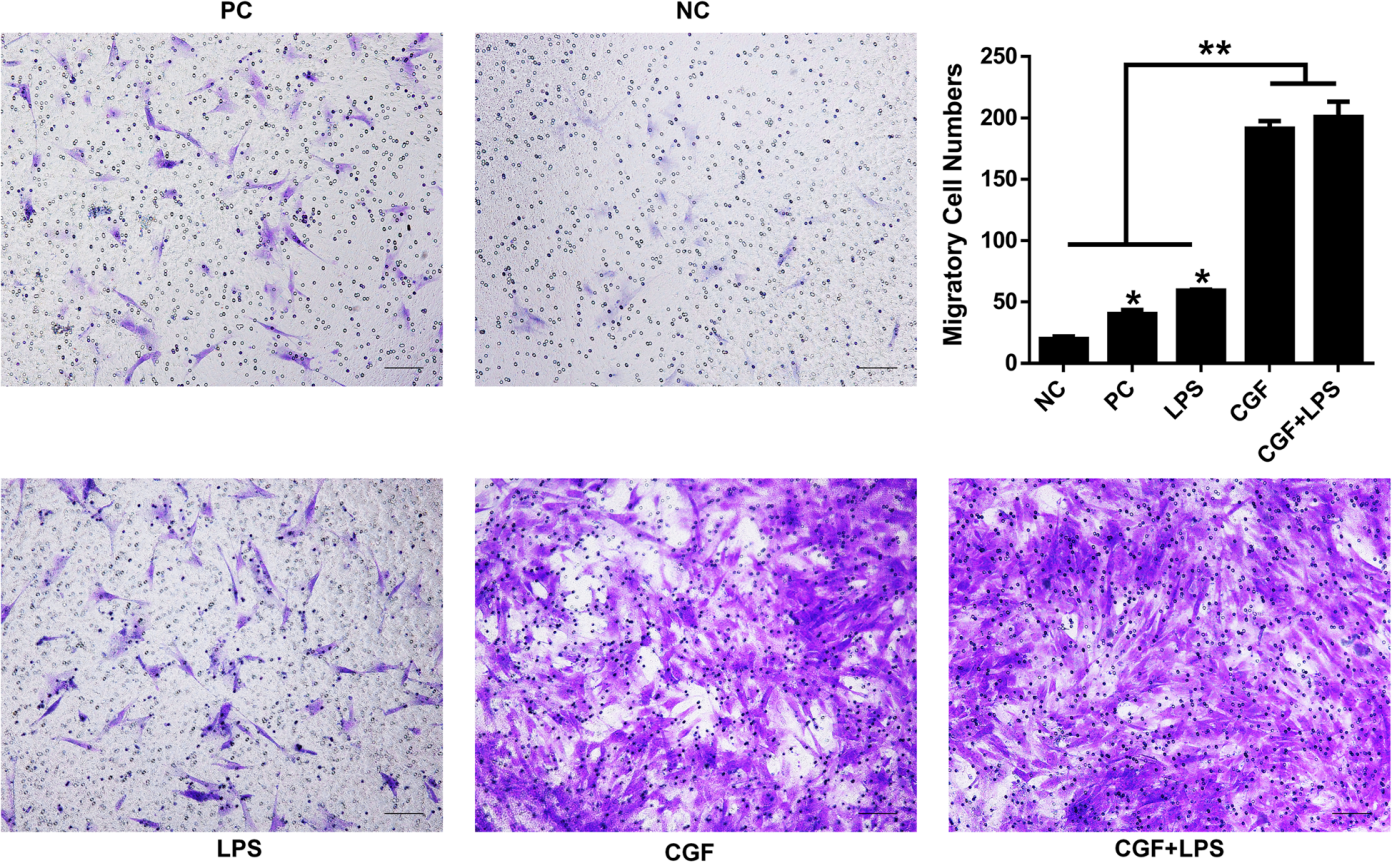
Effects of CGF on cell migration in LPS-stimulated hDPSCs. The crystal violet staining of the migratory cells from different groups after culture in 1× CGF with or without the presence of 1 μg/mL LPS for 24 h. Representative images were taken using a microscope (magnification × 200). Statistical analysis of the average migratory cell numbers per field from different groups at 24-h time point (*P < .05). NG, negative group (normal medium without serum); PG, positive group (normal medium with 10% serum); LPS, 1 μg/mL LPS treated group; CGF, 1 × CGF-conditioned medium; CGF+LPS, 1× CGF with 1 μg/mL LPS-stimulated group. Scale bar = 100 μm

To investigate the effect of CGF on the migration capacity of LPS-stimulated hDPSCs, cells were treated with 1**×** CGF with or without the presence of 1 μg/mL LPS for 24 h for transwell assay (Fig. 5). The results showed that the migratory cells in 1× CGF group with or without LPS presence were significantly denser than those in the control group (*P* < .05), and migratory cells in the LPS group were more than those in the positive serum group (Fig. 5).

### The effect of CGF on the differentiation in LPS-stimulated hDPSCs

To investigate the effect of CGF on the odonto/osteogenic capability of LPS-stimulated hDPSCs, cells were treated with or without 1**×** CGF in the presence of 1 μg/mL LPS for 4 and 7 days for ALP analysis and 21 and 28 days for alizarin red staining and quantitative polymerase chain reaction analysis.

**Fig. 6:**
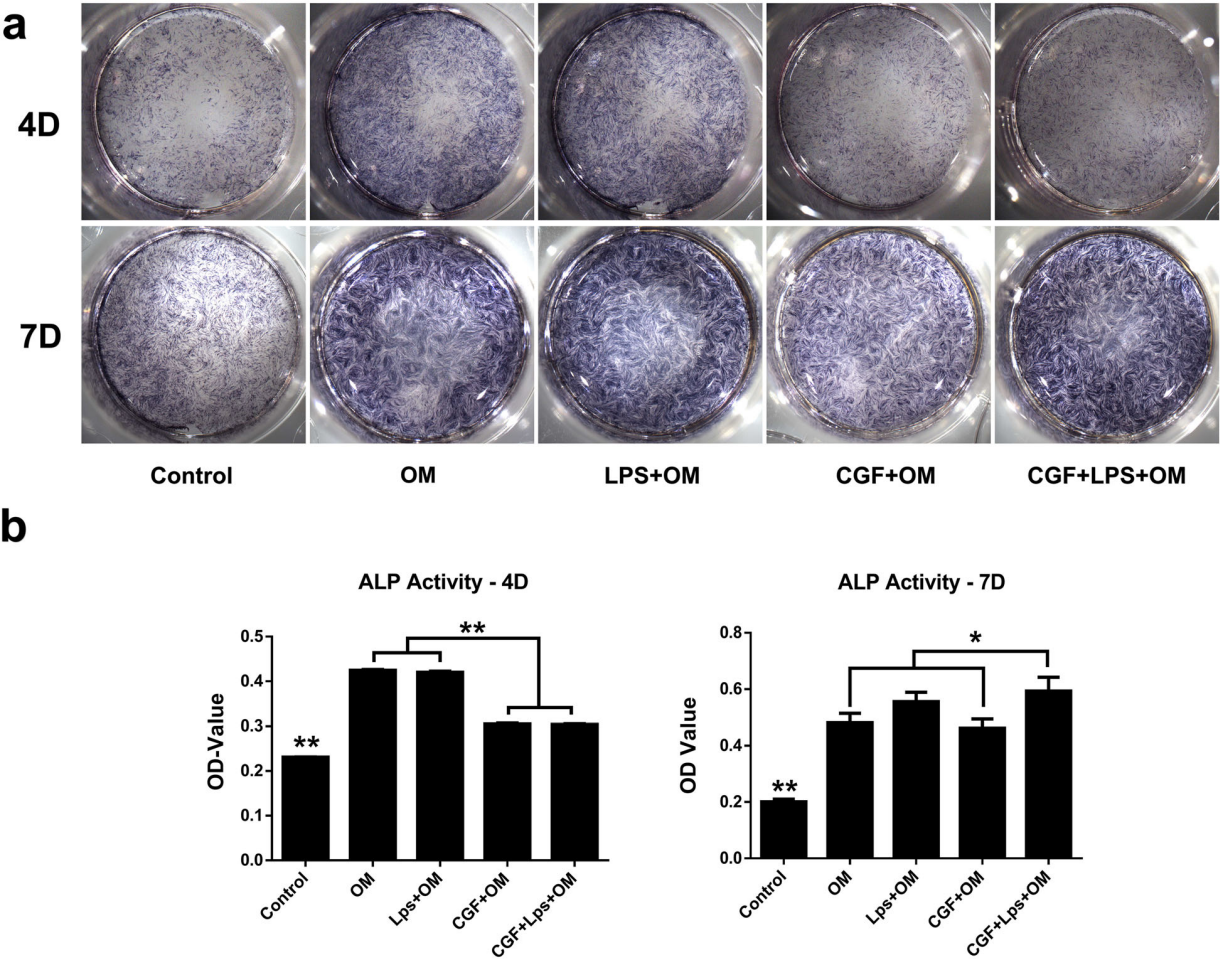
The effects of CGF on ALP activity of LPS-stimulated hDPSCs at different time points. Staining (**a**) and quantitative detection (**b**) of ALP activity in hDPSCs under incubation of 1**×** CGF or in the presence of 1 μg/mL LPS for 4, 7 days. The results were expressed as the means ± standard deviation of triplicate measurements from three independent experiments (*P < .05). Control, normal medium with 10% serum; OM, osteogenic-induced medium group; LPS**+**OM, 1 μg/mL LPS with the osteogenic-induced medium group; CGF**+**OM, 1**×** CGF-conditioned with the osteogenic-induced medium group; CGF+LPS, 1**×** CGF and 1 μg/mL LPS with the osteogenic-induced medium group

At day 4, osteogenic-induction medium (OM) with or without LPS could promote ALP activity of the hDPSCs as compared with the negative control group; however, 1**×** CGF suppressed this process under osteogenic induction with or without LPS (Fig. 6a). At day 7, 1**×** CGF significantly promoted the ALP activity of LPS-stimulated hDPSCs as compared with OM and LPS group (*P* < 0.05), LPS could also enhance the ALP activity of hDPSCs (Fig. 6a). These results were inconsistent with the quantitative ALP results (Fig. 6b).

**Fig. 7:**
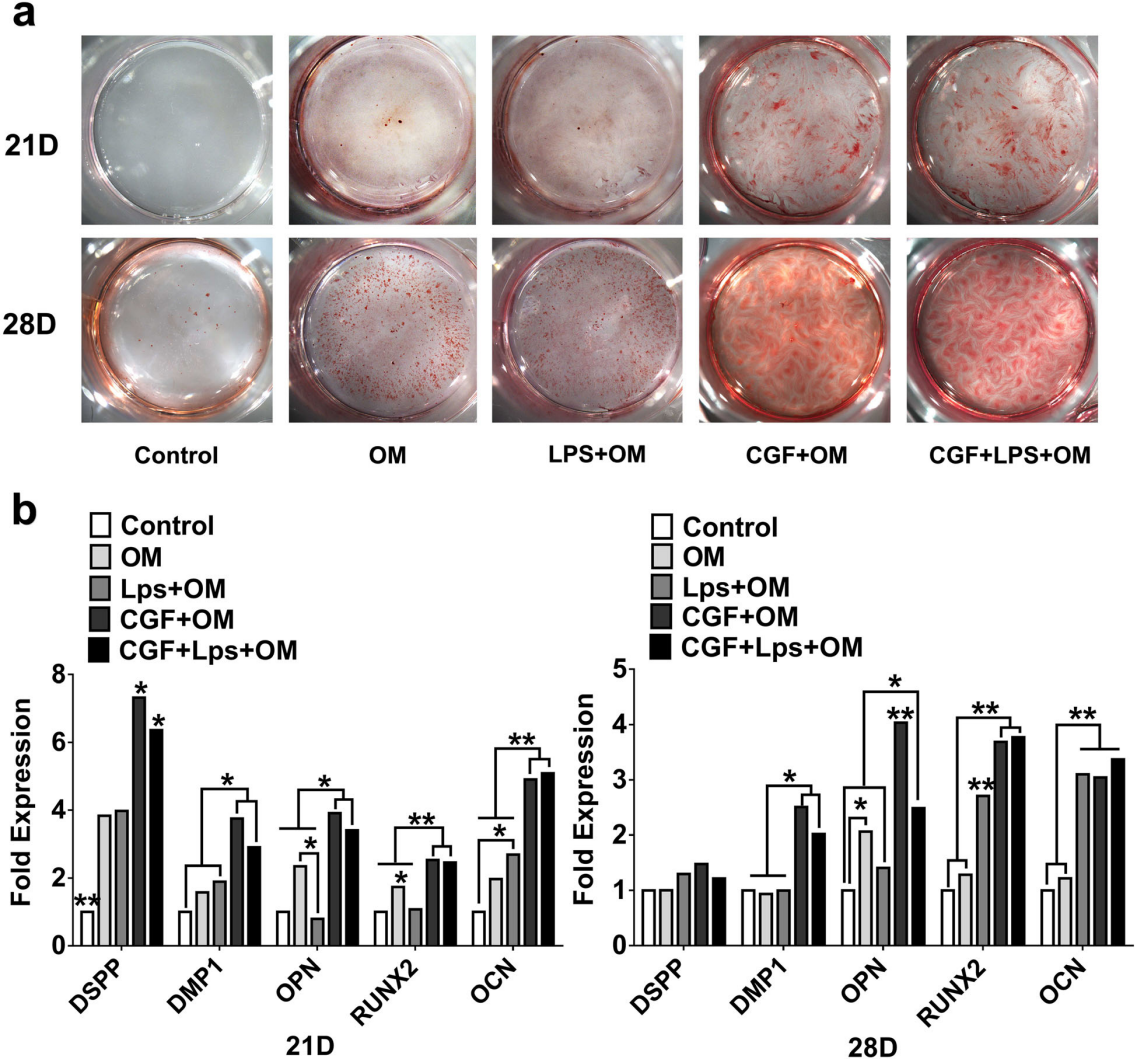
The effects of CGF on alizarin red staining and differentiation-related genes expression in LPS-stimulated hDPSCs. **a** Alizarin red staining of the hDPSCs after culture in 1× CGF with or without 1 μg/mL LPS treated for 21 days and 28 days. Representative photographs of calcified nodules stained with alizarin red S are shown. **b** Relative gene expression levels of DSPP, DMP-1, OPN, Runx2, and OCN of the hDPSCs after culture in 1× CGF with or without 1 μg/mL LPS treated for 21 days and 28 days (*P < .05, **P < .01). Control, normal medium with 10% serum; OM, osteogenic-induced medium group; LPS+OM, 1 μg/mL LPS with the osteogenic-induced medium group; CGF**+**OM, 1**×** CGF-conditioned with the osteogenic-induced medium group; CGF+LPS, 1× CGF and 1 μg/mL LPS with the osteogenic-induced medium group

Alizarin red staining showed that the mineralized areas were significantly increased by the co-treatment of LPS and CGF at day 21, and the staining gets stronger at day 28 (Fig. 7a). The gene expression levels of DSPP, DMP-1, OPN, Runx2, and OCN were greatly upregulated after incubation in CGF for 21 and 28 days (*P* < .05) (Fig. 7b). As compared with the LPS group, the gene expression of DSPP, DMP-1, OPN, Runx2, and OCN were significantly increased in the co-treatment of LPS and CGF group at day 21. Under LPS-stimulated condition, CGF could also enhance the mRNA expression of DMP-1, OPN, and Runx2 as compared with that in the LPS group at day 28 (Fig. 7b).

### The effect of CGF on the generation of dentine-pulp complex and the development of apical foramen of the immature teeth in the beagle dog

**Fig. 8:**
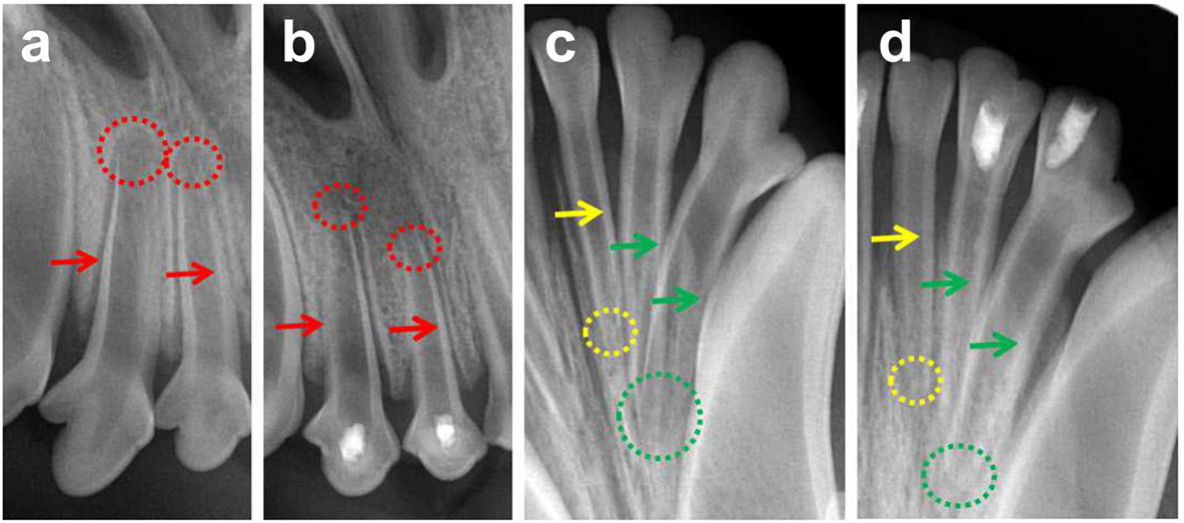
The radiographic results of CGF on the generation of dentine-pulp complex and the development of apical foramen of the immature canine teeth. **a**, **c** The radiographic images of the pre-operative teeth. **b**, **d** The radiographic images of the postoperative teeth after 8 weeks. Red arrowhead and dotted circle indicated the root canal walls and the apical foramen of the negative control group (pulp removed and left with no filling material) respectively. Yellow arrowhead and dotted circle indicated the root canal walls and the apical foramen of the positive control group (normal teeth) respectively. Green arrowhead and dotted circle indicated the root canal walls and the apical foramen of the CGF filling group respectively

The radiographic images revealed the presence of an open apex and the absence of preexisting pathosis in the pre-operative canine teeth (Fig. 8a, c). The positive control group exhibited a normally thickened root canal wall and a closed apex (yellow arrowhead and dotted circle in Fig. 8d). Meanwhile, after 8 weeks of the operation, the root canal wall (green arrowhead in Fig. 8d) of the CGF group presented different levels of root thickening as compared with the images before the operation (green arrowhead in Fig. 8c). As similar to the positive control group, the CGF group also showed a closed apex (green dotted circle in Fig. 8d). On the contrast, the root canal walls of the negative control group (pulp removed and left no filling material inside the root canal) (red arrowhead in Fig. 8b) showed no significant changes as compared with the images before the operation (red arrowhead Fig. 8a); however, the apical foramen of the negative control group was almost closed as well (red dotted circle in Fig. 8b).

### Histologic results

**Fig. 9:**
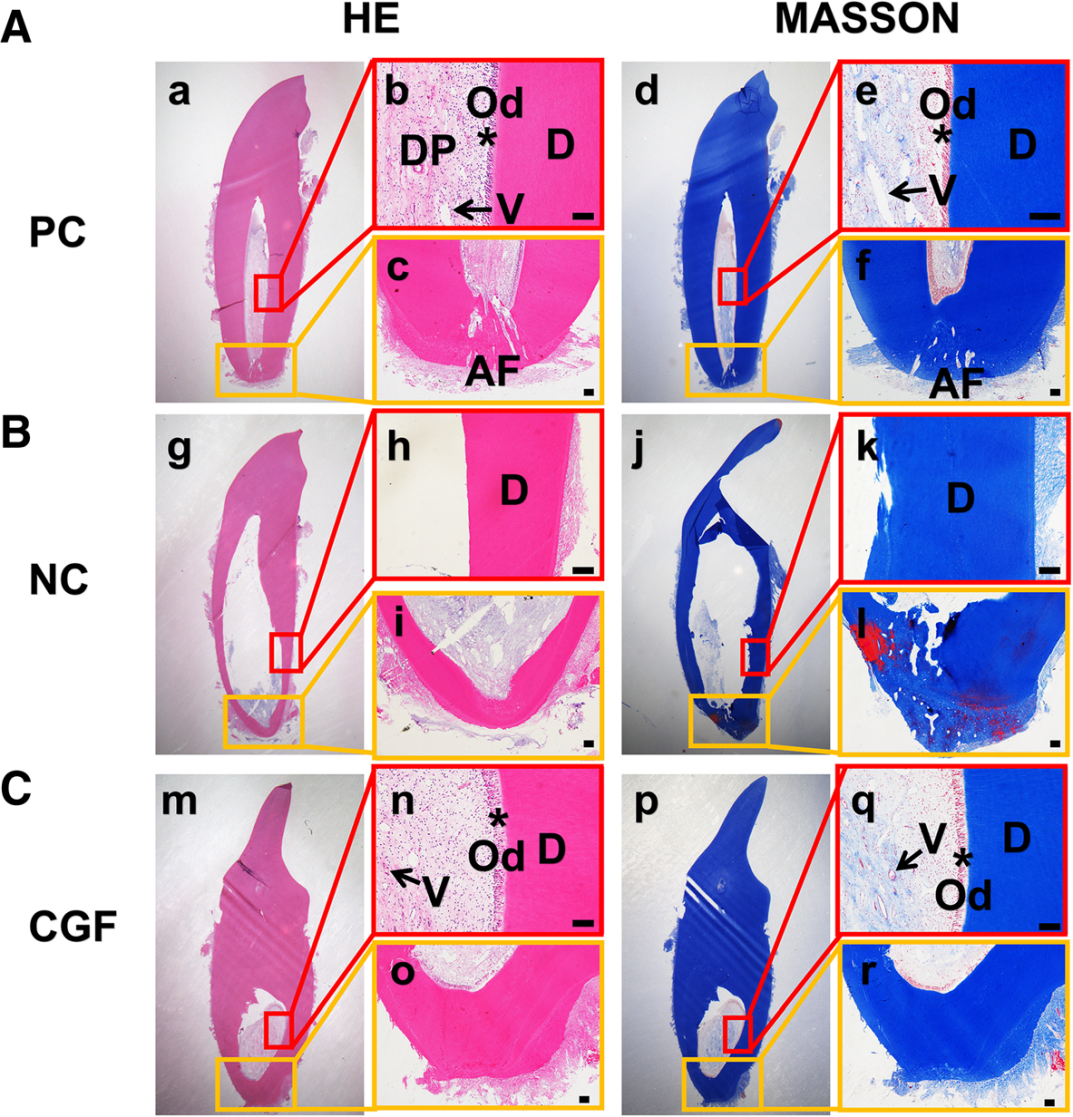
The histological results of CGF on the generation of dentine-pulp complex and the development of apical foramen of the immature canine teeth. The HE and Masson trichrome staining results of the positive control group (**A**), the negative control group (**B**) and the CGF filling group (**C**). **b**, **c**, **e**, **f**, **h**, **i**, **k**, **l**, **n**, **o**, **q**, **r** The amplified images of the square box in **a**, **d**, **g**, **j**, **m**, and **p**. Arrowhead indicates the vessels; the asterisk indicates the odontoblasts. DP, dental pulp; AF, apical foramen; V, vessel; Od, odontoblast; D, dentin. Scale bar = 100 μm

In the positive control group, we could see the thickened root canal wall and the closed apical foramen. Inside the root canal, there were normal pulp tissues which have numerous pulp cells, scattered blood vessels, and the surrounding odontoblasts (Fig. 9b, e). In the CGF group, there also exists a histological evidence of hard-tissue deposition in the internal root dentin wall (Fig. 9m, p) and an apical closure (Fig. 9o, r); the root canal was filled with the regenerated connective tissues with different degrees of similarity to normal dental pulp (Fig. 9n, q), which also has the palisading-arranged odontoblasts which were adjacent to the newly formed pre-dentin (asterisk in Fig. 9n, q). Besides, the scattered blood vessels and cerulean stained collagen fibers could also be seen in the central area of the regenerated pulp-like tissues (arrowhead in Fig. 9n, q). In the negative control group, the results exhibited empty lumina with no regenerated tissues and a closed apical foramen (i.e., no wall thickening or ingrowth of soft tissues into the root canal)(Fig. 9B).

**Fig. 10:**
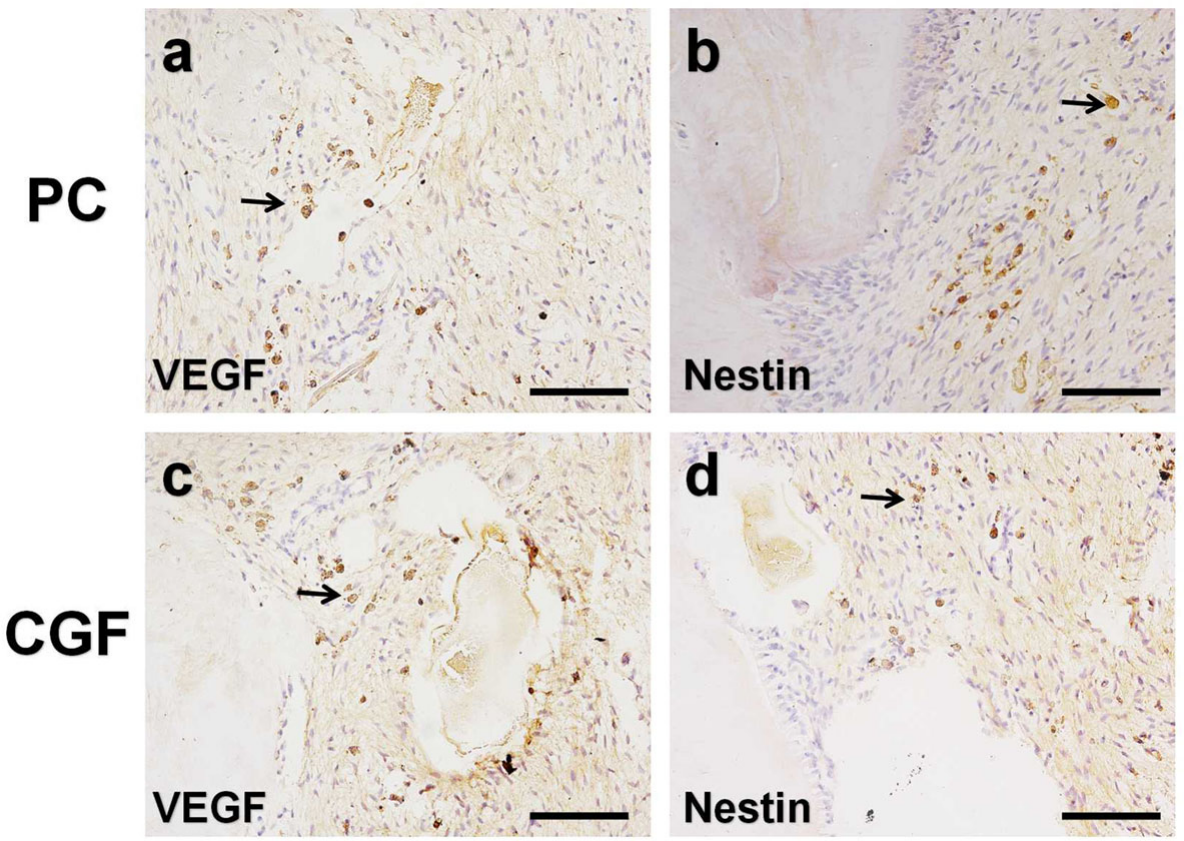
The effect of CGF on the vascularization and innervation in the soft tissues of the immature canine teeth. The immunohistochemical staining of VEGF (**a**) and Nestin (**b**) in the positive control group. **c**, **d** The VEGF and Nestin staining in the CGF filling group respectively. Arrowhead indicates the intense positive staining. Scale bar = 100 μm

IHC was performed for VEGF and Nestin in the newly formed pulp-like tissues in the CGF group and the positive control group (Fig. 10). The results revealed that the positive immmunoreactivity for VEGF in the CGF group was obvious throughout the regenerated soft tissues (Fig. 10c), which was similar to the positive staining in the normal pulp tissues (Fig. 10a), but it was particularly intense in the perivascular areas (arrowhead in Fig. 10a, c). The overall moderate Nestin staining was also seen in the regenerated pulp-like tissues, and it was particularly intense in the nerve-like cells (arrowhead in Fig. 10b, d). Negative controls for IHC using normal rabbit or mouse IgG never showed positive reactions in any sections examined (data not shown); we examined three to four different blocks for each sample and obtained the same results.

## Discussion

Dental pulp is a highly specialized loose connective tissue that preserves the teeth. A healthy dental pulp is not only responsible for tooth vitality, but also for pain sensation, immune defense, and tissue repair/regeneration after tooth injury. The major clinical injury to the teeth is bacterial penetration through caries lesions [22]. Several most common bacteria associated with dental caries are gram-negative bacteria. And LPS, as a main component of this bacterial membrane, is the prime toxic factor that contribute to the bacterial-induced immune response [10, 11]. According to the previous reports, LPS in the infected root canals was detected approximately ranging from 0.001 to 2 μg/mL [23, 24]. The method they used was by directly collecting samples through a paper point in the root canals and then quantified by limulus amebocyte lysate assay. However, the sampling they collected may not obtain all LPS molecules in the root canals. Therefore, the virtual concentration of LPS in the infected root canals might be higher than that in these reports. In order to better simulate the in vivo inflamed microenvironment in our in vitro research, we selected the concentrations of 1 μg/mL LPS on the basis of it was well used in many studies and could induce the biological responses of many mesenchymal stem cells [25, 26].

LPS can induce the expression of proinflammatory cytokines and chemokines, such as TNF-α, IL-6, and IL-8, and elicit a variety of immune responses in the odontoblasts, fibroblasts, and monocytes of dental pulp tissue [27–29]. Different doses and times of these proinflammatory cytokines may yield different effects in dental pulp cells. Chronic exposure (> 3 days) of dental pulp cells to TNF-α and IL-1β impairs its ability to differentiate into odontoblasts, suggesting that inflammatory cytokines may inhibit the repair and regeneration of dentin and pulp during inflammation [30]. Shorter and appropriate exposure of inflammatory molecules induced by TNF-α and LPS to dental pulp cells can upregulate the odontoblastic-related gene expression of DSPP and DMP-1 [31, 32]. In accordance with the previous study [33], our results showed that different doses of LPS can stimulate the expression of several inflammatory cytokines including IL-6, IL-8, IL-1β, and TNF-α in hDPSCs at days 1, 3, 5, and 7, which are the representative proinflammatory molecules detected in the inflamed pulp tissue [34]. As a member of the CXC chemokine family of cytokines, IL-8, along with TNF-α, can not only mediate the migration of neutrophils but also promote the recruitment of tissue stem cells to the injury sites and contribute to tissue healing [35–37]. The high detection of IL-8 in CGF may suggest its ability to recruit DPSCs that resided in distant pulp to the injury site. The initial inflammatory period is important for recruiting leucocytes and surrounding connective tissue cells for tissue healing. On the other hand, feedback signaling from the cells surrounding the injury site modulates the activation of resident macrophages by secretion of anti-inflammatory factors. As one of the most important proinflammatory cytokines, TNF-α can not only participate in vasodilatation and regulation of blood coagulation, but also contribute to increasing the synthesis of anti-inflammatory factors, such as IL-10 [38]. Therefore, CGF may increase the expression of TNF-α in LPS-stimulated hDPSCs at day 1 as the result of initial regulated inflammatory response. In addition, the expression of TNF-α was significantly decreased by CGF in LPS-stimulated hDPSCs at day 5, we speculated that these may result from the highly promoted cell proliferation by LPS. The enhanced cell proliferation can accelerate the process of tissue regeneration and wound healing and therefore inhibit the expression of TNF-α at day 5 [39]. According to Chiche et al.’s report at 2017 [40], IL-6 have a promoted effect on cellular reprogramming via senescent cells in the context of tissue injury, indicating us that IL-6 may have a positive role in tissue repairment. These may explain why the treatment of CGF did not inhibit the expression of IL-6 at days 3, 5 and 7. Meanwhile, CGF can attenuate the expression of IL-8 under LPS-stimulated condition from day 1 to day 7, indicating that CGF may have an inhibitory effect on the inflamed dental pulp cells and play a positive role in tissue repair.

In tissue regeneration strategies, an ideal scaffold is specifically designed to promote adhesion, proliferation, migration, and/or differentiation of the incorporated cells [41]. CGF, as an autologous biomaterial, has been used in oral, maxillofacial, plastic, and bone surgery as well as in gingival tissue engineering research [15, 16, 42]. As similar to our previous study that CGF can make the proliferation and migration of SCAPs elevated [17], in this study, CGF was revealed to promote the proliferation and migration of hDPSCs whether under LPS stimulated or not. Although LPS alone was found to promote the proliferation and migration of hDPSCs, the upregulation level of CGF in LPS-stimulated hDPSCs was much higher, indicating CGF can still accelerate the proliferation and migration of hDPSCs under LPS-stimulated condition. Previous studies have reported that proinflammatory cytokines can be secreted by a range of cells including odontoblasts in response to bacterial secreted LPS, and those proinflammatory cytokines were accounted for the migration of neutrophil and stem cells [43]. Meanwhile, CGF contains a variety of platelet cytokines and growth factors, including PDGF-BB, TGF-β1, and VEGF, which were critical growth factors participating the regulation of the proliferation of various cells [44, 45]. The previous study has reported that bFGF, chemotactic factors released from the CGF, had the same effect of migration on DPSCs as compared with G-CSF in vitro [46]. And PDGF-BB can also facilitate the migration of hDPSCs besides from their enhanced proliferation and odontoblastic differentiation ability [47]. Therefore, these chemotactic factors released from the CGF may play a vital role in pulp regeneration through mediating the inflammation and enhancing the proliferation and migration of the LPS-stimulated hDPSCs.

The critical step of pulp regeneration is the differentiation into odontoblasts and the formation of new dentin and capillaries. The odonto/osteoblastic differentiation of hDPSCs may be identified by detection of ALP activity and the mineralized nodule formation and the expression of several odonto/osteogenic genes, such as DSPP, DMP-1, Runx2, OPN, and OCN. In the present study, we analyzed ALP activity, which is considered as early markers of hard tissue formation or osteoblastic/odontoblastic differentiation. Co-treatment of CGF and LPS resulted in suppressed ALP activity and mineralization in hDPSCs when compared with the LPS and OM group on day 4. And at day 7, the co-treatment of CGF and LPS upregulated the ALP activity in hDPSCs. In order to examine the odonto/osteoblastic capacity of CGF in LPS-stimulated hDPSCs at late stages, the alizarin red staining was used to reveal the mineralized nodules in the LPS/CGF. LPS and CGF groups were denser and larger than those in the control group after incubation for 21 days and 28 days. At the same time, the co-treatment of LPS and CGF also increased the mRNA expression of DSPP, DMP1, Runx2, and OCN as compared with the control and LPS group although it resulted in no significant increase in DSPP, DMP-1, and OPN when compared with the CGF group. These results were in accordance with earlier finding in which LPS promoted the odontoblastic differentiation of hDPCs by increased mineralized nodule formation and gene expression of odontoblastic markers [48]. These results indicated that the CGF could significantly promote the odonto/osteogenic differentiation of the LPS-stimulated hDPSCs at the late stage.

The criteria for evaluating successful pulp regeneration was the regenerated pulp-like tissues should be connective tissues that (i) deposit new dentine, (ii) show similar cell density and architecture to the natural pulp, and (iii) have vascularization and (iv) innervation [49]. The results of in vivo study indicated that CGF could induce the thickened formation of dentin walls and the ingrowth of soft connective tissues which included the palisading arranged odontoblasts and the scattered blood vessels. As we know, CGF is a three-dimensional network that consists of the cross-linked fibrins, platelets, and various growth factors; those growth factors can not only recruit the stem cells residing in periapical areas such as stem cells from apical papilla (SCAPs), but also promote the proliferation and differentiation of those dental mesenchymal stem cells. Meanwhile, the natural-fibrin-like structure of CGF could also enable stem cells to grow better. VEGF plays an important role in angiogenesis by promoting endothelial cell proliferation, increasing vascular permeability, and altering the biological effects of extracellular matrix [50]. Nestin is associated with the pluripotency of neural stem cells and is expressed in both precursors of neurons and glial cells [51]. The IHC results of VEGF and Nestin indicated that CGF could regenerate the pulp-like tissues that resemble the natural one which has vascularization and innervation.

## Conclusion

The results of this study revealed that CGF can not only inhibit proinflammatory cytokines release and promote proliferation, migration, and odonto/osteogenic differentiation in the LPS-stimulated hDPSCs in vitro*,* but also promote the regeneration of dentine-pulp complex and achieve the continued development of the immature teeth in the beagle dog in vivo. Therefore, as a good combination of biomaterial and abundant growth factors and chemotactic factors, CGF can serve as a promising biomaterial due to its excellent regulatory properties in inflammation, proliferation, migration, and odonto/osteogenic differentiation to promote pulp regeneration in clinical pulp injury applications.

## Abbreviations

ALP: Alkaline phosphatase
bFGF: Basic fibroblast growth factor
CGF: Concentrated growth factor
DMP1: Dentin matrix protein 1
DSPP: Dentinsialophosphoprotein
hDPSCs: Human dental stem pulp cells
IGF-1: Insulin-like growth factor-1
IL: Interleukin
LPS: Lipopolysaccharide
OCN: Osteocalcin
OM: Osteogenic-induction
OPN: Osteopontin
PDGF-BB: Platelet-derived growth factor-BB
Runx2: Runt-related transcription factor 2
SCAPs: Stem cells from apical papilla
SCAPs: Stem cells of the apical papilla
TGF-β1: Transforming growth factor β-1
TNF-α: Tumor necrosis factor-alpha
VEGF: Vascular endothelial growth factor
